# The Impact of COVID-19 Pandemic on Italian University Students’ Mental Health: Changes across the Waves

**DOI:** 10.3390/ijerph18189897

**Published:** 2021-09-20

**Authors:** Micaela Di Consiglio, Sheila Merola, Tiziana Pascucci, Cristiano Violani, Alessandro Couyoumdjian

**Affiliations:** Department of Psychology, Sapienza University of Rome, 00185 Rome, Italy; micaela.diconsiglio@uniroma1.it (M.D.C.); sheila.merola@uniroma1.it (S.M.); tiziana.pascucci@uniroma1.it (T.P.); cristiano.violani@uniroma1.it (C.V.)

**Keywords:** COVID-19, university students, mental health, psychological distress, help-seeking behavior

## Abstract

To reduce the spread of COVID-19, the Italian government imposed a rigid lockdown and, for a whole year, continued to declare stringent rules to curb the community spread. This study provides an overview of university students’ symptomatology and help-seeking behaviour before and during the pandemic. It aims to evaluate the impact of the different phases of the pandemic on students’ mental health. We collected data in four-time points between March 2019 and March 2021. A total of 454 students (F = 85; M = 15) were included in the study. Students answered a socio-demographic and a standardized questionnaire (i.e., SCL-90-R) to evaluate a broad range of symptomatology. The results suggest that students experienced moderate to severe levels of depressive, obsessive-compulsive and anxiety symptomatology. About 14% of the sample met the criteria for at least one mental health disorder, but most were not receiving mental health care. During the lockdown, compared with other phases, female students reported worse symptoms in the obsessive-compulsive, interpersonal sensitivity, depression, paranoid ideation, and psychoticism dimensions. The increasing symptomatology disappeared after the lifting of the lockdown. The results showed no difference in the male groups. Preventive and support strategies should be improved in the university context.

## 1. Introduction

On 5 January 2020, the World Health Organization (WHO) issued the first disease outbreak news report about a severe acute respiratory syndrome cluster of unknown causes. Later, the WHO assessed that this disease, named COVID-19 and caused by coronavirus SARS-CoV-2, constituted a public health emergency of international concern, and could be characterized as a pandemic. As of March 2020, Italy was the second country globally in terms of registered cases and the first in terms of victims. To combat the rapid escalation of cases in Italy and curb the community spread, Italy’s government declared a state of emergency. The first and most rigid containment measure imposed was a national quarantine or lockdown, restricting the movement of the population except for necessity and health circumstances. Italy was the first state in Europe to follow such lockdown measures: attending school and going to work was not allowed, except for well-grounded work-related reasons, and public gatherings were prohibited. The decree also provided the obligation to stay isolated at home for anyone infected [1].

The lockdown caused a sudden change in the population’s habits and free movements. Consequently, mental health problems, including anxiety, fear, depressive symptoms, loneliness, and sleep problems, increased to some degree [2,3,4]. For example, a literature review that evaluated the effects of the COVID-19 pandemic on the population’s mental health showed high rates of different symptomatology such as anxiety (ranging from 6.33% to 50.9%), depression (ranging from 14.6% to 48.3%) and posttraumatic stress disorder (ranging from 7% to 53.8%). Moreover, the prevalence of psychological distress ranged from 34.43% to 38% [5]. Studies conducted on the potential psychological impact on the Italian general public have showed that, during the lockdown, a high prevalence of individuals presented anxiety and depressive symptoms, posttraumatic stress symptomatology and sleep disturbances [6,7,8]. Furthermore, some non-governmental organizations registered an alarming rise in the death rate by suicide; between March and November 2020, 100 out of 200 suicides and suicide attempts in Italy were correlated with COVID-19 [9]. Hence, in the last year, a number of studies were conducted to explore the effect of the lockdown on mental health, suggesting a significant negative impact on individuals’ health. However, even if the most stringent lockdown lasted some months, the Italian government continued to declare a series of containment measures to curb the community spread (Table 1). These measures changed quickly based on different indexes regarding the incidence rate, transmission numbers, hospital occupancy and other factors to assess the risk level in each region. Regions were classified into three areas —red, orange, or yellow—corresponding to three risk scenarios, for which specific restrictive measures were foreseen. Besides the three areas, the nation as a whole had to observe a night curfew (from 10 p.m. to 5 a.m.) and people were constantly obliged to wear a mask, including outdoors, and maintain a distance of at least one meter from other people [10]. To the best of our knowledge, less is known about the impact of such restrictions on individuals’ mental health.

### 1.1. Mental Health among University Students

The COVID-19 outbreak has impacted almost all sectors of society, including higher education. Indeed, all classes were suspended because of social distancing, and students had to follow lessons using online platforms during the lockdown [11]. These changes had a significant impact on students’ lifestyle, academic performance, and mental health. Most students have negative perceptions about e-learning and believe that it does not greatly impact their learning [12]. Approximately 50% of students reported a decrease in study hours and their academic performance, over 10% of students delayed graduation or dropped out from classes, about 40% of working students lost their job [13], and approximately 55% of students reported increasing concern about the exam outcomes [14]. Regarding mental health issues, students reported an increased level of stress, anxiety, and depression during the COVID-19 pandemic [15,16,17] and an increase in suicidal thoughts [18]. Moreover, students reported difficulty concentrating on academic work and negative changes in their sleep and dietary patterns [17]. Some studies found significant sex differences: female students showed more anxiety than male students [19], and they were at more risk of developing depression in comparison to males. Moreover, females reported more sleep and sexual problems [18]. Some factors contributed to increased stress, anxiety, and depression among university students, such as worry about health, disruption of the daily routine, decreased social interaction [17], and a history of self-injury and suicidal attempts [18]. These results are particularly relevant if we consider that, even before the pandemic, mental health problems were widespread among university students. Anxiety disorders (i.e., panic disorder, generalized anxiety disorders, and social phobia), mood disorders, and substance disorders are the most prevalent disorders among university students [20]. Even if it is not a specific diagnosis, suicide is a significant problem among university students: 6.7% of students have suicidal ideation [21]. Moreover, mental health problems were associated with role impairment in different domains [22] and university career problems [23]. Often, students do not ask for psychological help despite feeling the need for it. Young people prefer to ask for help from friends or family rather than doctors or psychologists [24]. The general misinformation about mental health and the fear of being stigmatized frequently prevent help-seeking behaviour [25,26].

### 1.2. The Study: Aims and Scope

Such evidence suggests the need to prioritize students’ mental health [27] and that the psychological health of university students impacted by the pandemic should be taken seriously. Sapienza University of Rome has offered online psychological support to its students in the wake of this emergency. Online therapy represents the best way to help students facing psychological and emotional problems due to the pandemic and the consequence of routine disruption [28]. In addition to a counselling service, students could also join the NoiBene program. NoiBene is a web-based intervention to promote psychological well-being and prevent psychological distress by developing a series of competencies (i.e., life skills) and reducing dysfunctional coping strategies. Before the pandemic, NoiBene had already been used, and analyses showed its effectiveness in promoting psychological well-being and reducing dysfunctional coping strategies among university students [29]. To deal with COVID-19-related stress, modules about loneliness, relaxation techniques, breathing exercises, and mindfulness were added. Moreover, the intervention included individual weekly meetings with a tutor, a psychologist that supervised the program. The tutor aimed to monitor the online program’s progress, provide answers to any questions that the students might have about the exercises, and give support for any issues concerning the quarantine.

The present study aims to provide an explorative and descriptive overview of the symptomatology and help-seeking behaviour of students included in NoiBene between March 2019 and March 2021. The Ethical Committee of the Department of Psychology, Sapienza University of Rome, approved the NoiBene protocol. We hypothesized that the following restrictions had impacted the individuals’ psychological health as well as the first quarantine. We divided students into three phases: (a) March 2020–May 2020 (Quarantine Phase); (b) October 2020–December 2020 (Second Phase); (c) January 2021–March 2021 (Third Phase). The quarantine phase was characterized by the most stringent lockdown and by a gradual reopening. During the second phase, after an increase in transmission, the government imposed new restrictive measures such as the mandatory use of masks (including outdoors), the prohibition of gatherings of more than a certain number of people, and a nationwide night curfew. Moreover, the government classified regions into three areas corresponding to three risk scenarios. Lastly, during the third phase, besides previous rules that were maintained, a specific national quarantine was imposed during the national holidays (Table 1). Therefore, we compared clinical symptomatology between students Before COVID with students gathered in the three phases (Quarantine Phase, Second Phase, Third Phase). We formulated a series of research hypotheses based on the specific restriction measures in the different phases, the evolution of the pandemic, and the increasing knowledge about COVID-19 (including the origin of the virus, its transmission, and the mechanisms to stop its spread).

Considering that the COVID-19 quarantine significantly limited social interaction, thereby increasing feelings of loneliness [30,31], we expected that students’ depressive symptomatology and relational problems during the first quarantine were higher than those of students in the other groups. Moreover, considering the high level of uncertainty regarding health and economic issues during the quarantine and the uncertainty about the evolution of the pandemic, we expected that anxiety symptomatology during the first quarantine was higher than anxiety symptomatology during the other phases [32]. Lastly, we expected that students’ distress was higher than the Second Phase but lower than the Quarantine Phase during the Third Phase. Indeed, in the Third Phase, people brought with them a year of restriction and suffering. Moreover, the beginning of the vaccination campaign promoted hope and a more positive perspective of change but, on the other hand, was associated with fear and uncertainty regarding its efficacy [33,34].

## 2. Materials and Methods

### 2.1. Participants

NoiBene was publicized through the official Sapienza website on a page devoted to promoting well-being services. Participation was voluntary and free of charge. NoiBene was presented as a guided self-help program to develop some useful skills to cope with the well-being challenges brought about by the pandemic. Informed consent about the research protocol was presented to every student that asked to follow the program. Twenty-one students did not accept to be included in the study, so they used the program, but their data were excluded from the analysis. The final sample was composed of four-hundred-and-fifty-four (*n* = 454) students, aged from 19 to 54 (M = 24.80; SD = 4.10), with a majority being female (M = 68; F = 386). Most of the students live with their parents (63.1%), with flatmates (25.3%) and a minority with their partners (5.7%), alone (3.7%) or with a brother or sister (1.1%). The majority of participants (61.7%) were enrolled in the Faculty of Medicine and Psychology and were completing a Master’s degree course (59%). About 35.9% of the students never sought psychological help, 15.9% had accessed mental health treatment in the past, and 6.8% were in ongoing therapy. About 41.4% did not give this information.

### 2.2. Measures

To assess psychological distress, we administered, to every student, the Symptom Checklist-90-Revised, SCL-90-R [35,36], a multidimensional self-report inventory covering nine dimensions of psychological distress: somatization (SOM—distress arising from bodily perceptions), obsessive-compulsive (OC—thoughts, impulses, and actions that are experienced as irresistible), interpersonal sensitivity (IS—feelings of personal inadequacy and inferiority, and distress during interpersonal interactions), depression (DEP—symptoms of depressive syndromes), anxiety (ANX—symptoms that are associated with manifest anxiety), hostility (HOS—thoughts and feelings of anger, irritability, and resentment), phobic anxiety (PHOB—persistent fear response to a specific person, place, object, or situation that leads to avoidance or escape behaviour), paranoid ideation (PAR—projective thinking, hostility, suspiciousness, and centrality), and psychoticism (PSY—withdrawal, isolation, and schizoid lifestyle). It also included three global indices of psychological distress: the Global Severity Index (GSI—index of overall psychological distress), the Positive Symptom Distress Index (PSDI—index of the intensity of symptoms), and the Positive Symptoms Total (PST—number of self-reported symptoms). The scores were converted to standard T-scores using the norm group appropriate for the participants. T-scores between 55 and 65 suggested moderate to elevated symptomatology; T-scores above 65 suggested elevated symptoms. The Symptom Checklist-90-Revised is an established instrument and has several studies supporting its reliability and validity. Its test-retest reliability has been reported at 0.80 to 0.90 with a time interval of one week. All nine primary subscales correlate with other broad-range inventories [35]. The Italian translation and validation showed Cronbach α values from 0.68 to 0.87 for the nine dimensions [36]. In our study, the Cronbach α ranged from 0.77 to 0.91 for the nine dimensions. 

Moreover, every student completed an ad hoc questionnaire to collect demographic data (i.e., age, occupation, residence) and information about their academic status (i.e., faculty, degree course). 

### 2.3. Procedure

Every student that asked to participate received a personal account on NoiBene [29] and provided informed consent about data protection and privacy according to the General Data Protection Regulation (GDPR; EU 2016/679). Then, they answered the SCL-90-R questionnaire. Students with elevated levels of symptomatology were contacted for a diagnostic interview. If severe ongoing clinical conditions (e.g., mood disorders, psychotic disorders) or suicidal ideation were confirmed, the student received feedback about their symptomatology and was directed towards the treatment suited to their needs. In this case, NoiBene was used as a support for their therapy. Otherwise, each student was contacted by a tutor. An experienced psychotherapist supervised the activity of the tutors during the duration of the program. Students could meet the tutor once a week; the meetings were held on video-call platforms, guaranteeing a private space. Every meeting started with a mood check. Then, the tutor introduced specific contents regarding psychological well-being or cognitive vulnerability and discussed any issues with the students. Between meetings, every student was asked to complete the module regarding the topic discussed previously. Indeed, except for the first one, every meeting included a revision about homework (see Table S1 in Supplementary Materials for more details).

## 3. Statistical Analyses

Statistical analyses were performed using SPSS version 27.0 for Windows (IBM Corp., Armonk, NY). A series of descriptive analyses were conducted on participant characteristics (i.e., demographic variables, academic data, symptomatology, and help-seeking behaviour). A Chi-Squared Test was run to examine whether there was a difference between groups in the proportion of male and female participants. It showed a significantly different distribution of males and females across groups. For this reason, group comparison analyses were conducted considering males and females differently. A series of one-way analysis of variances (ANOVAs) were conducted to investigate differences between four female groups (BeforeCOVID vs. Quarantine vs. SecondPhase vs. ThirdPhase). Considering that the males’ symptomatology rating was non-normally distributed, as measured by the Shapiro–Wilk test (*p* values ranged from <0.001 to 0.02), a series of non-parametric Kruskal–Wallis analyses were conducted to investigate differences between four male groups (BeforeCOVID vs. Quarantine vs. SecondPhase vs. ThirdPhase). The level of significance was set at *p* < 0.05. Effect sizes were calculated using partial eta squared (partial- η^2^) for ANOVAs and eta squared (η^2^) for Kruskal–Wallis analyses. Both were interpreted based on benchmarks suggested by Cohen [37]: η^2^ = 0.01, small effect size; η^2^ = 0.06, medium effect size; η^2^ = 0.14, large effect size. The post hoc analyses of significant interactions were conducted using the Fisher LSD post hoc test.

## 4. Results

### 4.1. Preliminary Analyses 

To investigate any differences between groups in terms of the age and gender variables, we conducted a one-way ANOVA and a Chi-Squared test, respectively. No significant differences were found for the age variable (*F*(3, 453) = 0.58, *p* = 0.63). A Chi-Squared test showed a gender difference between groups (χ^2^ (23, *N* = 454) = 12.8, *p* = 0.005). In particular, the Adjusted Standardized Residual (ASR), an index based on the difference between the observed counts and expected counts, suggests a significant number of males in the Quarantine group (ASR = 2.8) and a significant number of females in the Third group (ASR = 2.4). Considering the different distribution of male and females across groups (Table 2), we decided to conduct group comparison analyses differently for the male and female groups.

### 4.2. Symptomatology 

As shown in Table 3, students’ mean scores were below the pathological cut-off in every clinical dimension. However, it is noteworthy that the percentage of students who scored above the cut-off was considerable. For example, about 35.2% of students reported an elevated level of depressive symptomatology, and 32.5% of students reported obsessive-compulsive symptomatology. Moreover, about 28.6% of students presented an elevated level of psychological distress and 32.8% of students reported intensive symptomatology. 

### 4.3. Group Comparison 

A series of one-way ANOVAs were conducted to investigate differences between four female groups: BeforeCOVID vs. Quarantine vs. SecondPhase vs. ThirdPhase. As shown in Table 4, one-way ANOVA showed a significant difference between groups on obsessive-compulsive symptomatology (*F*(3, 385) = 3.51, *p* = 0.015, partial-η^2^ = 0.03). A further post hoc test found that participants in the Quarantine group scored higher than participants in BeforeCOVID group (*p* = 0.005), in the SecondPhase group (*p* = 0.002) and in ThirdPhase group (*p* = 0.014). Results indicated a significant difference between groups in interpersonal sensitivity symptoms (*F*(3, 385) = 5.11, *p* = 0.002, partial-η^2^ = 0.04). A further post hoc test found that feelings of personal inadequacy and inferiority were significantly higher in the Quarantine group than in BeforeCOVID group (*p* < 0.001), in the SecondPhase group (*p* < 0.001) and in ThirdPhase group (*p* < 0.001). Analyses showed a significant difference between groups on depression symptomatology (*F*(3, 385) = 4.20, *p* = 0.006, partial-η^2^ = 0.03). Post hoc analyses suggest that the level of depression in the Quarantine group was statistically higher than the BeforeCOVID group (*p* < 0.001), SecondPhase group (*p* = 0.006) and higher than the ThirdPhase group (*p* = 0.004). There was a statistically significant difference between groups in the paranoid ideation dimension as determined by one-way ANOVA (*F*(3, 385) = 3.76, *p* = 0.011, partial-η^2^ = 0.03). Post hoc analyses suggest that hostility and suspiciousness thoughts in the Quarantine group were statistically higher than BeforeCOVID group (*p* = 0.014), SecondPhase group (*p* = 0.002) and statistically higher than ThirdPhase group (*p* = 0.003). Moreover, analysis showed significant differences in the psychoticism dimension (*F*(3, 385) = 3.96; *p* = 0.008, partial-η^2^ = 0.03). A post hoc test revealed that withdrawal and isolation behaviours, in the Quarantine group were statistically higher than the BeforeCOVID group (*p* = 0.01), the SecondPhase group (*p* = 0.005) and statistically higher than the ThirdPhase group (*p* < 0.001). One-way-ANOVA showed a significant difference between groups on the level of psychological distress (*F(3, 385)*= 3.65, *p* = 0.013, partial-η^2^ = 0.03). Post hoc analysis indicated that the level of overall psychological distress in the Quarantine group was significantly higher than the BeforeCOVID group (*p* = 0.004), SecondPhase group (*p* = 0.004) and ThirdPhase group (*p* = 0.004). Lastly, analysis showed a significant difference between groups on the number of self-reported symptoms (*F*(3, 385) = 4.41, *p* = 0.005, partial-η^2^ = 0.03). Post hoc analyses indicate that the number of self-reported symptoms in the Quarantine group was statistically higher than the BeforeCOVID group (*p* < 0.001), SecondPhase group (*p* = 0.003) and statistically higher than the ThirdPhase group (*p* = 0.002). There were no statistically significant differences between group means as determined by one-way ANOVA in the other dimensions. See Figure S1 in Supplementary Materials for a graphic representation of descriptive analyses.

A series of Kruskal–Wallis analyses were conducted to investigate differences between four male groups: BeforeCOVID vs. Quarantine vs. SecondPhase vs. ThirdPhase. As shown in Table 5, the results showed no significant differences between the male groups. See Figure S2 in Supplementary Materials for a graphic representation of descriptive analyses.

## 5. Discussion

The present study aims to provide an explorative and descriptive overview regarding the psychological distress and symptomatology of students included in NoiBene between March 2019 and March 2021. Moreover, we investigated whether the different phases of the COVID-19 restrictions impacted students’ mental health differently. 

### 5.1. Participant Characteristics

Most of the sample was composed of females. These data were consistent with the percentage of females usually included in web-based interventions [38] and with data suggesting that males are less disposed to seek mental help than women [39]. Interestingly, more males asked to participate in NoiBene during the lockdown compared with other phases. From the beginning of the pandemic, it was immediately apparent that, apart from physical health, mental health needed to be seriously taken into consideration. For this reason, psychologists and non-governmental services increased and strengthened online counselling therapy, or e-therapy [40]. It is possible to hypothesize that the increased attention to mental health has normalized the need to ask for help. Considering that the perceptions of normativeness influence help-seeking behaviour [41], this could be the reason why a significant number of males asked for help during the quarantine.

### 5.2. Symptomatology and Help-Seeking Behavior

Our data revealed that many students experienced moderate to severe levels of depression symptomatology, obsessive-compulsive symptomatology, and anxiety symptomatology. After completing the first screening, one-hundred-and-thirty-two students (29.07% of the total sample size) were contacted for a psychodiagnostic interview due to the high scores obtained. Of the 132 students, 24.2% students refused the interview. A broad range of evidence identified a series of barriers to treatment such as low perceived need, the desire to handle the problem on one’s own, attitudinal and structural barriers [42] and internalized and treatment stigma [43]. Considering that these students decided to participate in NoiBene, we can hypothesize that they perceived a need, but they wanted to handle the problem independently. Indeed, young people often use self-help programs, such as NoiBene, to deal with their mental health problems [24]. One hundred students accepted the interview. Of the 100 students, 64% students met the criteria for at least one mental disorder according to The Diagnostic and Statistical Manual of Mental Disorders, DSM-5 [44]. Of these students, eleven (*n* = 11) were already receiving mental health care, so they were included in the NoiBene program. In contrast, after the interview, fifty-three students (*n* = 53) were directed toward psychotherapy. Of the 53 students, about 55% of students decided to start psychotherapy, but twenty-three (*n* = 24) did not accept. This is a remarkable outcome: thanks to the individual meeting that we conducted, we had the opportunity to give personalized feedback about the individual’s symptomatology and inform students about how and where to find help in the area. According to the high percentage of students that accepted psychological therapy, it seems to be an efficacious strategy [45]. Regarding students that did not accept, we had the opportunity to explore individual barriers to help-seeking during the interview. The main reasons for refusing were the low perceived need, the fear of being misunderstood by other relevant people, or the idea that talking with a psychologist would exacerbate their problems. Besides the low perceived need, the students’ choices were influenced by the treatment stigma, that is, the stigma associated with treatment for mental health, and the anticipated stigma, in other words, the fear of being perceived unfairly by others [43]. Nevertheless, these students asked for access at NoiBene, suggesting that NoiBene reached people who would otherwise not ask for help. Future research should use this advantage and focus on developing a strategy to reduce the stigma among students who are reluctant to start psychotherapy.

### 5.3. Group Comparison

Our findings partially supported the first hypothesis of our study: only female students showed an increase in depression and anxiety symptomatology and increasing feelings of inadequacy, inferiority, hostility, suspiciousness, and isolated lifestyle. Despite the significant effect, the effect size ranged from a small to medium, suggesting that these differences are not robust. For this reason, the results should be carefully interpreted. As a pandemic is an extraordinary event that cannot be replicated, in interpreting this finding, we should consider that other additional factors could explain the differences found between groups. For example, we have to keep in mind that every group is composed of different students asking to participate at different times of the year. For this reason, we should consider both variables related to students’ university commitments, such as exams and examination sessions, and other variables, such as coping strategies to deal with the different containment measures. Even if only tentative interpretations can be suggested given the small to medium effect size, it is possible to hypothesize, looking at the results as a whole, that during the quarantine, the most affected areas were related to mood and the quality of interpersonal relations. Indeed, this pattern of symptomatology is recurring in patients with major depression [46]. Moreover, it is possible to hypothesize that social distancing contributed to the arising of interpersonal relation uncertainties and the presence of negative expectations about interpersonal relationships. In addition, since COVID-19 spreads mainly between people who are in close contact with each other, it could have contributed to raising the perception of the other as a risk for one’s health, resulting in hostility or paranoid behaviour. Previous studies, which focused mainly on intimate relationships during the lockdown, indicated that high-stress levels were associated with a decline in intimate relationships [47]. Moreover, the attachment style of partners predicts interpersonal problems and the efficacy of problem-solving strategies [48]. Other studies suggested that working at home can exacerbate familial conflict [49] and that living with others contributed to increased psychological distress [50]. Interestingly, it seems that quarantine did not have an influence on male students. The results showed no significant differences between male groups. These data are consistent with other studies suggesting that COVID-19 had a more negative impact on females than males [51]. Some evidence suggested that during the lockdown, females reported higher levels of stress [50] and anxiety [19] than males and that they were at more risk of developing depression compared to males [18]. Lastly, our findings did not support our second hypothesis. The results did not show a significant increase in psychological distress in the Third Phase. However, despite the non-significant difference between groups, the males’ descriptive statistics show that students reported more anxiety and depressive symptoms, hostility and suspiciousness, and overall psychological distress during the Third Phase. Furthermore, even though the difference was not statistically significant, the effect size ranged from small to medium. The small male sample size may have prevented sufficient power to detect differences between groups. Therefore, the findings observed in this study need to be clarified by increasing our sample size. Moreover, to the best of our knowledge, less is known about the longitudinal changes in males’ mental health since most studies focused on the gender differences in relation to mental health during COVID-19. Future studies should investigate variables that could affect the different mental health trajectories across the pandemic between males and females.

### 5.4. Limitations and Future Directions

The study has some limits. First, most of the participants were female. For this reason, we decided to run an analysis for males and females separately. However, the tiny male sample size could have increased the occurrence of Type II Errors, reducing the chance of identifying a significant difference that could exist. Future studies should focus on a strategy to bring males closer to the topic of mental health. Second, our sample recruitment was not totally random: students decided spontaneously to participate in the NoiBene program, which could suggest that they have a particular interest in improving their mental health or that they perceive a need for help. This could have contributed both to the high percentage of psychopathological students and to the heterogeneity of our samples. Even if these limits could have contributed to reducing the study’s generalizability, the different flow of students that asked to be included in NoiBene at different moments represent an essential indicator of help-seeking behaviour. Lastly, the four groups that we considered in our analysis were composed of different participants, so we did not have the opportunity to examine any changes over time. However, we had the opportunity to compare psychopathological dimensions between groups to understand how different phases of the COVID-19 restrictions impacted the individual’s psychological health. It would be interesting in future studies to differentiate between students living in different regions with different risk scenarios, or to take into account risk and protective factors that underly the psychopathology that occurred during the pandemic. 

Besides these limitations, the study has different strengths: we had the chance to assess students before and during the pandemic. This allowed us to observe any change that was associated with the quarantine and the following restrictive rules. Moreover, we conducted individual interviews with every student reporting a high level of psychological distress: this had a fundamental impact in terms of helping students to understand the best treatment according to their needs [45]. In addition, self-report questionnaires can yield much valuable and diagnostic information, but they cannot be used to define a diagnosis. Conducting individual interviews allowed us to go beyond this limit and be sure about students’ symptomatology.

## 6. Conclusions

This study indicates that the lockdown had a significant negative impact on female mental health. Despite the increasing symptomatology during the quarantine among female students, the results suggest that it quickly disappeared after the lifting of the quarantine. Overall, the present study provides new insights into the impact of the pandemic on students’ mental health and supports data about psychological distress among university students. Moreover, it gives new perspectives in the field of help-seeking behaviour. Awareness of these topics can be helpful to encourage universities to integrate mental health into their culture and implement preventive and support interventions.

## Figures and Tables

**Table 1:** Containment measures in Italy.

Phase	Date	Decree
Quarantine Phase (March–May)	9 March 2020	National lockdown (at-home quarantine, closure of non-essential businesses, schools, and universities).
	4 May 2020	People were allowed to visit their relatives.
	18 May 2020	Reopening of bars, restaurants, beauty centres and other commercial and non-essential businesses.
Second Phase (October–December)	8 October 2020	Mandatory use of masks.
	13 October 2020	Limits on gatherings.
	26 October 2020	Closure of sports centres, cinemas, theatres, museums, and other public spaces and gathering places.
	6 November 2020	Imposition of nationwide night curfew and classification of regions into three areas—red, orange, and yellow—corresponding to three risk scenarios, for which specific restrictive measures were foreseen.
	24–27 December 2020	National red zone.
Third Phase (January–March)	1–3 January 2021	National red zone.
	5–6 January 2021	National red zone.
	7 January 2021	Specific restrictive measures in every region based on the risk scenarios.

Source: https://www.gazzettaufficiale.it/ (accessed on 12 July 2021).

**Table 2:** Gender by group.

Gender	Before COVID (*n* = 153)	Quarantine (*n* = 74)	Second Phase (*n* = 98)	Third Phase (*n* = 129)	Total (*n* = 454)
F	82.4%	74.3%	88.8%	91.5%	85.02%
M	17.6%	25.7%	11.2%	8.5%	14.98%

**Table 3:** Mean scores (*SD*) of SCL-90-R and percentage of pathological students.

Variable	*M* (*SD*)	55 ≤ T < 65 (%)	T ≥ 65 (%)	Total (%)
SCL-90-R				
Somatization	48.84 (10.77)	12.6	11.0	23.6
Obsessive-compulsive	50.09 (11.39)	17.6	14.5	32.5
Interpersonal sensitivity	48.03 (9.94)	16.5	7.3	23.8
Depression	52.35 (12.27)	16.7	18.5	35.2
Anxiety	50.38 (10.72)	17.0	11.7	28.6
Anger-hostility	47.59 (8.65)	13.0	4.8	17.8
Phobic anxiety	50.14 (10.35)	12.6	8.6	21.1
Paranoid ideation	44.10 (8.47)	8.1	3.7	11.9
Psychoticism	49.95 (9.43)	14.1	9.7	23.8
GSI	49.66 (10.80)	16.7	11.9	28.6
PSDI	51.24 (9.77)	21.6	11.2	32.8
PST	48.18 (11.45)	18.5	11.2	29.7

SCL-90-R = Symptom Checklist-90-Revised; GSI = Global Severity Index; PSDI = Positive Symptom Distress Index; PST = Positive Symptom.

**Table 4:** Mean (*SD*) score by female groups; ANOVA analysis.

	Groups						
	Before COVID (*n* = 126)	Quarantine (*n* = 55)	Second Phase (*n* = 87)	Third Phase (*n* = 118)	*F* (*df* = 3)	*p*	Partial-η^2^
SCL-90-R							
Somatization	48.82 (10.89)	51.44 (9.75)	48.02 (11.22)	48.29 (10.74)	1.34	0.262	0.010
Obsessive-compulsive	48.70 (11.62)	53.74 (11.75)	47.97 (10.01)	49.31 (10.65)	3.51	0.015	0.027
Interpersonal sensitivity	47.10 (9.76)	52.37 (11.55)	46.64 (9.62)	46.72 (8.86)	5.11	0.002	0.038
Depression	50.34 (12.59)	57.01 (11.86)	51.37 (11.84)	51.37 (11.36)	4.20	0.006	0.032
Anxiety	49.49 (11.80)	52.28 (10.73)	49.07 (9.20)	49.84 (9.42)	1.21	0.305	0.001
Anger-hostility	47.25 (8.72)	49.67 (10.43)	47.25 (8.19)	47.24 (8.07)	1.23	0.300	0.010
Phobic anxiety	49.11 (9.53)	52.29 (13.68)	51.55 (11.49)	48.84 (7.33)	2.46	0.062	0.019
Paranoid ideation	43.63 (8.88)	46.91 (9.15)	42.55 (6.79)	42.86 (8.01)	3.76	0.011	0.029
Psychoticism	49.31 (9.87)	53.05 (10.95)	48.75 (7.89)	48.20 (7.27)	3.96	0.008	0.030
GSI	48.49 (11.65)	53.45 (10.58)	48.21 (9.88)	48.46 (9.53)	3.65	0.013	0.028
PSDI	46.46 (11.98)	52.56 (10.11)	46.89 (10.54)	46.94 (10.76)	2.20	0.088	0.017
PST	50.22 (10.20)	54.01 (9.45)	50.28 (10.11)	50.86 (8.95)	4.41	0.005	0.033

SCL-90-R = Symptom Checklist-90-Revised; GSI = Global Severity Index; PSDI = Positive Symptom Distress Index; PST = Positive Symptom.

**Table 5:** Mean (*SD*) score by male groups; Kruskal–Wallis analysis.

	Before COVID (*n* = 27)		Quarantine (*n* = 19)		Second Phase (*n* = 11)		Third Phase (*n* = 11)		Kruskal–Wallis		
	*M* (*SD*)	Mean Rank	*M* (*SD*)	Mean Rank	*M* (*SD*)	Mean Rank	*M* (*SD*)	Mean Rank	*X^2^* *(df* = 3)	*p* Values	η^2^
SCL-90-R											
Somatization	48.98 (10.91)	34.96	44.69 (6.19)	28.11	52.91 (19.69)	41.73	51.09 (11.23)	37.18	3.70	0.296	0.011
Obsessive-compulsive	52.07 (13.46)	30.96	50.69 (10.80)	29.76	56.82 (10.76)	40.55	60.45 (11.61)	45.32	6.29	0.098	0.051
Interpersonal sensitivity	51.33 (12.01)	35.02	48.89 (9.26)	31.71	48.55 (8.39)	32.14	51.73 (7.17)	40.41	1.54	0.672	0.023
Depression	54.11 (13.59)	32.72	53.13 (12.14)	31.18	55.55 (10.90)	35.50	61.27 (13.94)	43.59	3.11	0.374	0.002
Anxiety	52.52 (12.92)	31.94	49.66 (7.06)	31.45	52.82 (13.70)	33.36	60.82 (13.17)	47.18	5.50	0.139	0.040
Anger-hostility	47.22 (7.93)	33.89	47.92 (10.16)	34.21	45.09 (6.19)	28.68	50.36 (8.96)	42.32	2.73	0.434	0.004
Phobic anxiety	49.80 (11.31)	30.78	48.77 (7.92)	33.26	51.36 (9.07)	36.95	55.91 (17.10)	43.32	3.71	0.294	0.011
Paranoid ideation	48.72 (9.90)	38.07	44.08 (7.30)	28.53	43.55 (8.50)	26.55	50.09 (7.82)	44.00	7.01	0.072	0.063
Psychoticism	52.85 (12.82)	32.81	51.92 (8.35)	33.87	54.27 (14.43)	34.50	55.09 (9.18)	39.73	0.99	0.803	0.031
GSI	52.63 (13.57)	33.04	49.44 (8.59)	30.24	53.18 (11.38)	35.36	57.82 (9.57)	44.59	3.92	0.270	0.014
PSDI	51.61 (11.01)	30.83	48.38 (10.84)	34.39	50.77 (11.36)	39.77	58.05 (10.26)	38.41	2.14	0.543	0.013
PST	51.24 (11.28)	33.50	52.81 (8.71)	29.89	55.27 (10.60)	33.77	54.05 (7.11)	45.64	4.61	0.203	0.025

SCL-90-R = Symptom Checklist-90-Revised; GSI = Global Severity Index; PSDI = Positive Symptom Distress Index; PST = Positive Symptom.

## Data Availability

Group-level information about the data is available from the corresponding author on reasonable request.

## Supplementary Materials

The following are available online at https://www.mdpi.com/article/10.3390/ijerph18189897/s1, Table S1: Protocol of intervention, Figure S1: Mean (SD) score by female groups, Figure S2: Mean (SD) score by male groups.
