# Supplementary material for: The Impact of COVID-19 Pandemic on Italian University Students’ Mental Health: Changes across the Waves
Source: Int J Environ Res Public Health. 2021 Sep 20;18(18):9897. doi: 10.3390/ijerph18189897 (PMC8469053; doi:10.3390/ijerph18189897)
Supplement: Supplementary file 1 [file ijerph-18-09897-s001.zip › ijerph-1373182-supplementary.pdf]

International Journal of
Environmental Research and Public Health

MDPI

Table S1. Protocol of intervention.

Meeting	Goal and Activities	Homework
1st meeting	1. To introduce the program and to get to know each other.<br>2. To elicit commitment and motivation by asking the student to schedule a timetable of future access to the platform and to set the goal that they want to reach with the program.	Module: Commitment and motivation<br><br>The concepts of well-being and mental health are explained.
2nd meeting	1. Mood check<br>2. Psychoeducation about emotions, needs and values.	Module: Self-awareness<br><br>The aim is to help students become aware of their emotions and needs and to understand the personal thought and evaluation that underlie each emotion. Students are asked to use a personal diary designed to report one's own emotive episodes. Then, some exercises are proposed to help students identify their values system.
3rd meeting	1. Mood check<br>2. Homework revision<br>3. Introduction of cognitive vulnerabilities	Module: Psychological bugs<br><br>The aim is to help students to identify the presence of any psychological bugs (e.g. mental processes and attitudes that contribute to emotional suffering and relationship problems such as repetitive thinking, perfectionism, and experiential avoidance)
4th meeting	1. Mood check<br>2. Homework revision<br>3. Psychoeducation about rumination, worry and self-criticism.	Module: Repetitive thinking<br><br>The aim is to help students to challenge themselves with repetitive thinking. Students are asked to use a personal diary to monitor their repetitive thinking behavior. Then, students are encouraged to follow some techniques to challenge such thoughts.
5th meeting	1. Mood check<br>2. Homework revision<br>3. Psychoeducation about perfectionism.	Module: Perfectionism<br><br>The aim is to help students to challenge themselves with perfectionism. Students are asked to complete exercises to identify personal high standards and the positive and negative aspects for being a perfectionist. Then, students are encouraged to follow some techniques to challenge perfectionistic thoughts and behavior.
6th meeting	1. Mood check<br>2. Homework revision<br>3. Psychoeducation about avoidance and psychological inflexibility.	Module: Avoidance<br><br>The aim is to help students to challenge avoidance and psychological inflexibility. Students are asked to use a personal diary focusing on the situations that they tend to avoid. Students are encouraged to follow some techniques to challenge avoidance.
7th and 8th meeting	1. Mood check<br>2. Homework revision<br>3. Psychoeducation about the passive, aggressive and assertive style of communication.<br>4. The various steps to develop an assertive communication are presented.	Module: Social skills<br><br>The aim is to promote the development of healthy and positive relationships through the improvement of some fundamental social competences: active listening, assertive communication, and gratitude. Students are asked to complete some exercise and self-monitoring tools to identify their predominant communicative style and to develop an

	5. Psychoeducation about gratitude	assertive communicative style. Lastly, some exercise to increase gratitude are proposed (i.e. the gratitude diary and the gratitude letter)
9th meeting	1. Mood check<br>2. Homework revision<br>3. Introduction of the SMART model to reach personal goals. Different study methods are introduced, and students are helped to identify the best one that fits their needs and competences. Introduction of different mnemonics, such as the Loci Method or the Model Mnemonics.	Module: Self-realization<br><br>Students have to set a goal following the SMART model. Students are encouraged to set goals in accordance with their values (previously exposed in the Self-awareness module).
10th meeting	1. Mood check<br>2. Homework revision<br>3. Feedback	
Psychological First Aid		
The module "Psychological First Aid" is an always accessible module. It includes contents and exercises to face loneliness, rejection, failure, emotional loss. Moreover, it includes contents and exercises about anxiety and stress regulation, such as mindfulness and relaxation techniques, and about acceptance and distress tolerance. Lastly, it includes some sections about anxiety and depressive disorders: the aim is to help students identify the first signs of clinically relevant conditions and to promote help-seeking behavior.		

Figure 1. Mean (*SD*) score by female groups.

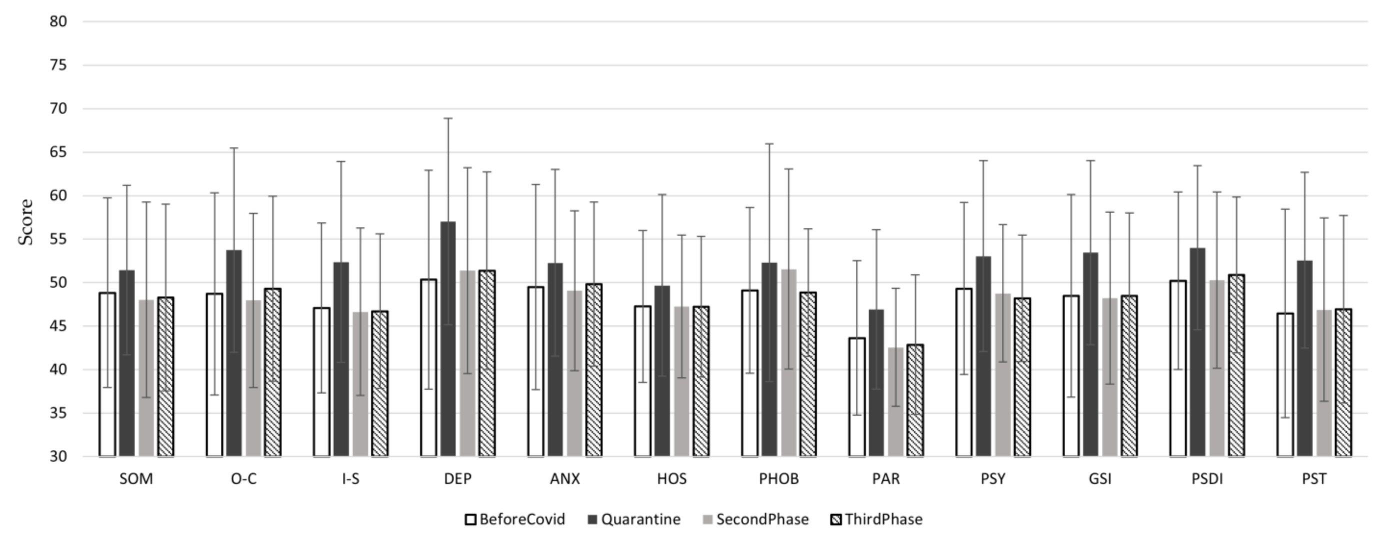

Figure S1. Mean (*SD*) score by female groups.

Figure 2. Mean (SD) score by male groups.

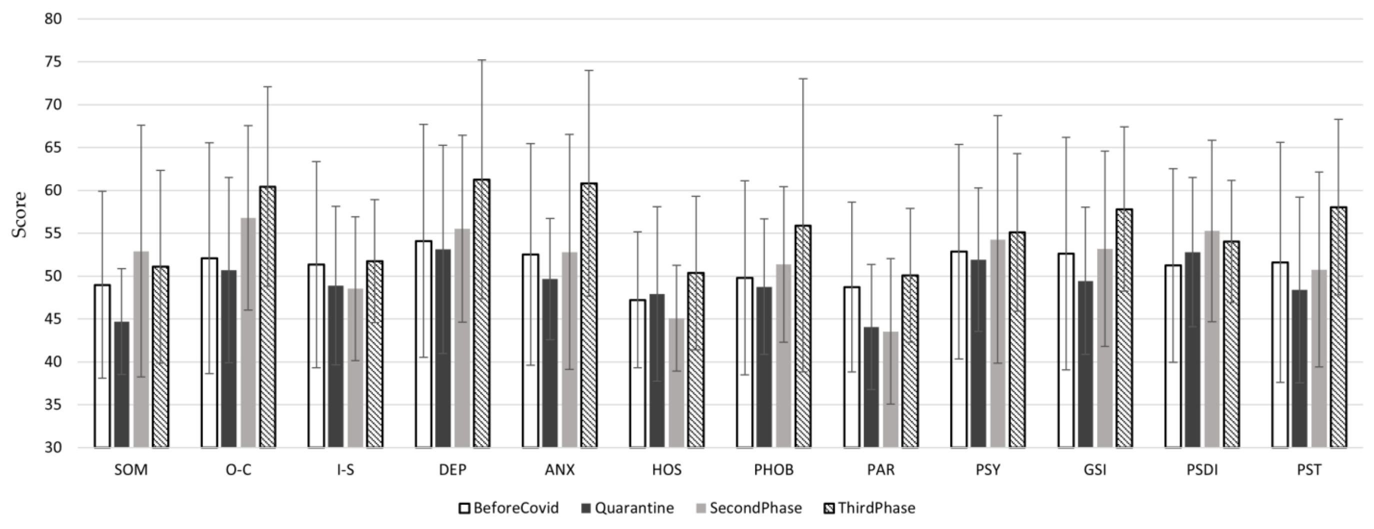

Figure S2. Mean (*SD*) score by male groups.
